# Hypertrophic angulation deformity of the basal interventricular septum combined with abnormality of the papillary muscle and chordae tendineae

**DOI:** 10.5830/CVJA-2016-050

**Published:** 2017

**Authors:** Yi Wang, Luwei Ye, Lixue Yin, Jie Zeng

**Affiliations:** Institute of Ultrasound Medicine, Sichuan Academy of Medical Sciences and Sichuan Provincial People’s Hospital, Chengdu 610072, China; Institute of Ultrasound Medicine, Sichuan Academy of Medical Sciences and Sichuan Provincial People’s Hospital, Chengdu 610072, China; Institute of Ultrasound Medicine, Sichuan Academy of Medical Sciences and Sichuan Provincial People’s Hospital, Chengdu 610072, China; Department of Cardiology, Sichuan Academy of Medical Sciences and Sichuan Provincial People’s Hospital, Chengdu 610072, China

**Keywords:** angulation deformity,, interventricular septum,, papillary muscle,, hypertrophic cardiomyopathy

## Abstract

A Chinese woman was admitted to our hospital because of syncope. Transthoracic echocardiography revealed a hypertrophic basal interventricular septum of 15 mm with a sharp angle protruding into the left ventricular outflow tract. Moreover, an anomalous anterolateral papillary muscle (maximum width of 11 mm) was inserted into the left ventricular outflow tract, with short chordae tendineae connecting both basal interventricular septum and anterior leaflet of the mitral valve. All of these abnormalities resulted in a left ventricular outflow gradient of 136 mmHg. Surgical septal myectomy of the sharp angle combined with partial papillary muscle resection and removal of the abnormal chordae tendineae was selected to relieve the left ventricular outflow obstruction. This was a rare combination of deformity of the angulation of the focal basal interventricular septum and abnormalities of the papillary muscle and chordae tendineae, which led to left ventricular outflow obstruction.

## Case report

**Fig. 1.**
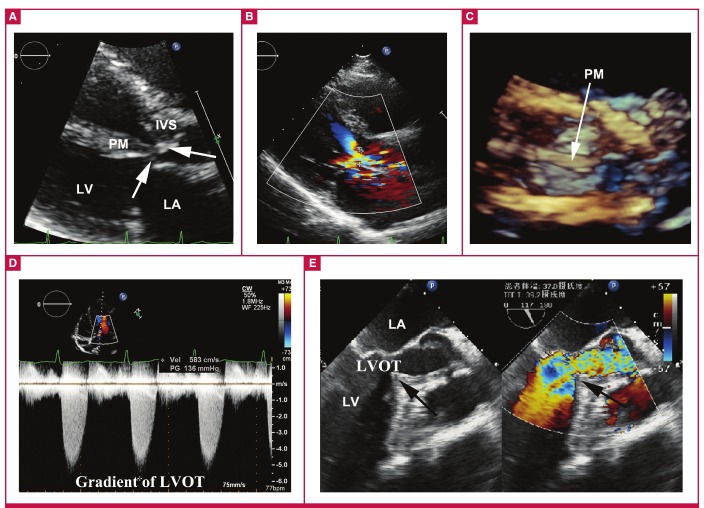
LVOT obstruction due to basal IVS hypertrophy (A) and PM malposition (A, C). B shows the narrowed blood flow of the LVOT. Pulsed Doppler demonstrated a gradient of 136 mmHg (D). Intra-operative transoesophageal echocardiography (E) showing the sharp angle of the IVS (black arrow).

A 43-year-old Chinese woman was admitted to our hospital because of syncope. Physical examination showed a loud systolic ejection murmur radiating to the neck. The electrocardiogram was normal. Transthoracic echocardiography revealed a hypertrophic basal interventricular septum (IVS) of 15 mm with a sharp angle protruding into the left ventricular outflow tract (LVOT). Moreover, an anomalous anterolateral papillary muscle (PM) (maximum width of 11 mm) was inserted into the LVOT, with short chordae tendineae connecting both the basal IVS and anterior leaflet of the mitral valve. All these abnormalities resulted in a LVOT gradient of 136 mmHg (Fig. 1A–D).

In order to verify the large gradient and exclude other associated abnormalities, cardiac catheterisation was performed. There was no stenosis of the coronary artery, but after the catheter was put into the left ventricle, it immediately went into the aorta, which indicated a large gradient of the LVOT. Left ventriculography also demonstrated a narrow LVOT with a sharp angle.

**Fig. 2.**
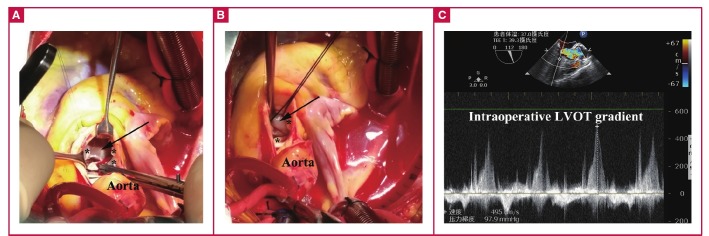
The sharp angle of the basal IVS (A, black arrow) and anomalous long PM (B, black arrow) were demonstrated through a transaortic approach (* aortic valve). After the first recovery of heart beat, transoesophageal echocardiography showed the LVOT gradient was still about 100 mmHg (C).

Surgery was selected to relieve the LVOT obstruction. Intraoperative transoesophageal echocardiography clearly showed the presence of a focal hypertrophic IVS and malposition of the PM (Fig. 1E). Through a standard median sternotomy, cardiopulmonary bypass was instituted by aortic/bicaval venous cannulation. After aortic cross-clamping, crystalloid cardioplegia solution was administered via the aortic root. The sharp angle of the IVS and the abnormal PM were demonstrated clearly after the extended transaortic approach was used (Fig. 2A, B).

The first step was septal myectomy of the sharp angle via the aortic valve. Partial PM resection was also performed to relieve the obstruction. Unfortunately, after recovery of the heart beat, transoesophageal echocardiography showed that the LVOT gradient was still about 100 mmHg because of the malposition of the PM and the short chordae tendineae (Fig. 2C). The surgeon then removed the abnormal chordae tendineae and the anomalous muscular attachments of the PM in the LVOT until a bougie of 20 mm passed through the LVOT smoothly. The saline injection test revealed trivial mitral and aortic valve regurgitation.

**Fig. 3.**
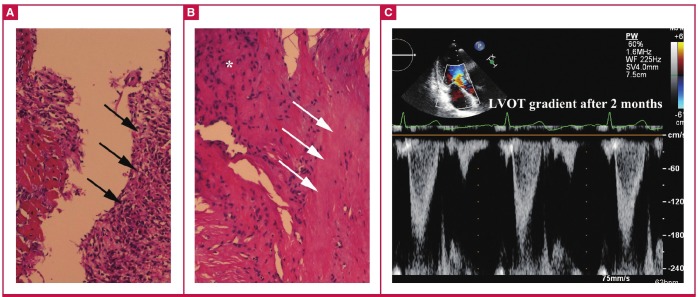
Histological examination showed inflammation (A, black arrows), cardiomyocyte hypertrophy and disarray (B, *), as well as interstitial fibrosis (B, white arrows) (haematoxylin and eosin, x100). At the two-month follow up, transthoracic echocardiography demonstrated a LVOT gradient of 23 mmHg (C).

Histological examination showed cardiomyocyte hypertrophy and disarray, as well as interstitial fibrosis and inflammation, indicating a possible diagnosis of hypertrophic cardiomyopathy (HCM) (Fig. 3A, B). Unfortunately, the patient was unwilling to do any genetic testing.

At the two-month follow up, transthoracic echocardiography demonstrated a LVOT gradient of 23 mmHg and no significant mitral valve insufficiency (Fig. 3C). In addition, the patient had no syncope or other uncomfortable symptoms. Institutional review board permission was obtained to report this case.

## Discussion

The cardiac phenotype of HCM shows great diversity in the degree and pattern of hypertrophy, such as asymmetric, concentric or apical.^1^ Asymmetric hypertrophy is often located in the whole IVS, not in a focal site. To the best of our knowledge, there has been only one case reported that revealed isolated posterobasal left ventricular free wall hypertrophy, which has extended the morphological diversity of HCM.^2^

In our case, the basal IVS not only showed hypertrophy, but also exhibited an angulation deformity, which has never been reported. The second interesting abnormality was the PM. It has been suggested that isolated PM hypertrophy is a possible variant of HCM, but only a few cases have been reported in the literature.^3^-^6^ Some of these patients presented with electrocardiographic findings, such as high left precordial voltage and inverted T waves, especially those with posteromedial PM hypertrophy.^3^,^5^ In our case, hypertrophy and malposition occurred at the anterolateral PM.

Treatment of HCM is based on the anatomical abnormality. In a subset of patients with HCM, LVOT obstruction will be present not only because of septal hypertrophy, but also owing to muscular apposition created by the abnormal PM. Failure to recognise this anomaly would not relieve the obstruction.^7^

In our case, aside from the abnormal PM and IVS, the chordae tendineae connecting them with the mitral valve also contributed to the crowded LVOT. Pre-operative identification of these three contributors to LVOT obstruction altered the surgical strategy. Therefore, this patient underwent septal myectomy of the sharp angle, partial PM resection in the LVOT, as well as removal of the abnormal chordae tendineae. Excessive PM resection could have caused mitral valve insufficiency, which may have resulted in the need for mitral valve replacement or surgical mitral leaflet manipulation. Fortunately, the saline injection test showed only trivial mitral valve regurgitation.

Redaelli et al.^8^ proposed a procedure to reposition the anterior PM followed by adjunctive implantation of a complete semi-rigid mitral ring to abolish the systolic anterior motion and residual mitral insufficiency. If our patient had had moderate to severe mitral regurgitation, a semi-rigid mitral ring or even mitral valve replacement would have been considered.

## Conclusion

Different mechanisms causing LVOT obstruction may occur in subgroups of patients with HCM. The mechanism of this rare LVOT obstruction resulted from focal basal IVS hypertrophy and angulation deformity, and abnormality of the PM and chordae tendineae. Although we did not have genetic evidence, this abnormal combination may represent a gap in our knowledge of HCM. Careful echocardiographic and other radiological assessment is needed before surgery, which could change the diagnosis and management of HCM.
